# Pain in emergency units: correlation with risk classification categories*

**DOI:** 10.1590/1518-8345.2415.3070

**Published:** 2018-11-14

**Authors:** Wandressa Letícia Viveiros, Meiry Fernanda Pinto Okuno, Cássia Regina Vancini Campanharo, Maria Carolina Barbosa Teixeira Lopes, Gabriella Novelli Oliveira, Ruth Ester Assayag Batista

**Affiliations:** 1 Universidade Federal de São Paulo, Escola Paulista de Enfermagem, São Paulo, SP, Brazil.; 2 Universidade de São Paulo, Hospital Universitário, São Paulo, SP, Brazil.

**Keywords:** Triage, Emergency Medical Services, Pain, Emergency Nursing, Pain Measurement, Nursing

## Abstract

**Objectives:**

to correlate risk classification categories with the level of pain of patients in an emergency service.

**Method:**

cross-sectional study carried out in the Risk Classification of 611 patients. The variables studied were: age, gender, comorbidities, complaint duration, medical specialty, signs and symptoms, outcome, color attributed in the risk classification of and degree of pain. We used Analysis of Variance, a Chi-Square test and a Likelihood Ratio test.

**Results:**

the average age was 42.1 years (17.8); 59.9% were women; the green (58.9%) and yellow (22.7%) risk classification prevailed and hypertension (18.3%) was the most common Comorbidity. The most frequent pain intensity was moderate (25.9%). In the red category, patients presented a higher percentage of absence of pain; in the blue, mild pain; and in the green, yellow and orange categories, there was a greater percentage of intense pain (p < 0.0001).

**Conclusion:**

among the patients who presented pain, the majority reported moderate intensity. Regarding risk categories, most patients in the red category did not report pain. Those who were classified as green, yellow and orange, reported mostly intense pain. On the other hand, patients in the blue category reported predominantly mild pain.

## Introduction

The situation of emergency services has been a matter of concern to the health community and to society. The demand for these services has increased due to high rates of urban violence, accidents and aging populations with a consequent increase of chronic diseases^1^ ^-^ ^2^. Furthermore, many of the cases received in emergency units are the result of low complexity diseases, referred to these services due to lack of structure in the basic health network, that could be resolved in basic or specialized units, or emergency services of lower complexity^3^. This demand profile characterizes the Emergency Service (ES) as one of the main entry points to the health system^1^.

The reception of patients with Risk Assessment and Classification (RRAC) was implemented to improve care in Emergency Services and consists of a system of initial evaluation of patients’ complaints with the main objective of providing care according to the level of severity, and no longer on a first-come, first-served basis^1^ ^,^ ^4^.

The RRAC performed by nurses is a tool to recognize patients who must be assisted within the shortest time possible. In the 1990s, several countries adopted and improved scales to classify patients’ risk. The most recognized international scales are: the Emergency Severity Index (ESI), the Australasian Triage Scale (ATS), the Canadian Triage Acuity Scale (CTAS) and the Manchester Triage System (MTS). In 2004, the Ministry of Health (MOH) created the QualiSUS Program and the National Humanization Policy (PNH), initiating the RRAC in Brazil as based on the Manchester protocol. The RRAC scales are different from each other and are often adapted to the places where they are used; yet, most of them rank patients into five risk categories, each category corresponding to a time interval which the patients can wait to receive medical attention, according to the severity of their situation^1^.

Among the health professionals authorized to conduct the risk classification process, after proper training, are the nurses. In a brief nursing consultation, the situation of the patient is evaluated through a physical examination focused on the complaints, personal history, and vital signs based on established protocols. After this process, the patient is informed about the estimated waiting time. When the complaint is painful, the intensity of the pain should be evaluated according to the protocol adopted at the institution^5^.

Pain is a symptomatic response of the organism, being a relevant sign at the moment of the evaluation. The search for emergency services is motivated by painful complaints which can be perceived in different ways. Thus, nurses must be aware of the time of their evaluation to provide the best care^6^.

Pain is defined by the American Pain Society as the fifth vital sign, and should be assessed along with temperature, respiratory rate, pulse and blood pressure. Its evaluation helps to diagnose the problem presented by the patients^6^. Nurses should investigate pain and use of instruments to assist in its measurement, as well as in the response to analgesia. Pain relief provides comfort and well-being to the individuals and promotes health during hospitalization or at home^7^.

Pain in the ES, in most cases, is acute and may be related to trauma or inflammatory processes^6^. Inadequate management of pain can cause problems such as increased blood pressure, heart rate and respiratory rate, resulting in worsening of the patients’ condition^6^.

Some obstacles have been identified in the evaluation of painful complaints of patients in Risk Classification, including the patients’ impaired ability to reliably report pain due to altered emotional state, anxiety due to the affected physical and mental state, and the type of approach by the professionals, because technical language sometimes makes it difficult for the patients to understand what is said. In addition, in ESs, there are many tasks to be performed in a short period of time as a result of the excessive flow of patients and need for fast care measures that can lead to an impaired evaluation of pain as a vital sign^6^.s

The management of pain in Emergency Services is complex because of its subjectivity, and still remains a challenge. The quality of safe and effective care can avoid complications resulting from prolonged pain, as well as provide the patient with greater comfort in the care in these places^8^.

The objective of this study was to correlate the risk classification categories with the level of pain of patients in an emergency service. The secondary objective was to correlate the degree of pain with sociodemographic variables, comorbidities, medical specialty, and signs and symptoms presented by patients who sought emergency care.

## Method

This is a cross-sectional study with quantitative analysis carried out in the sector of Risk Classification of the Emergency Service of the Hospital of São Paulo (HSP), a public university institution of high complexity located in the South Zone of São Paulo.

RRAC works 24 hours a day, seven days a week and is performed by nurses who make a brief nursing consultation in which the patients are classified and given colors according to severity categories. The RRAC protocol used at the Hospital of São Paulo was developed at the institution and is based on the protocol of the Ministry of Health, but uses five severity categories (colors)^1^. The colors used and the recommended times are: red (immediate care), orange (care up to 10 minutes), yellow (care up to 60 minutes), green (care up to 120 minutes) and blue (care up to 240 minutes); after classification, patients are referred to clinical (medical clinic, neurology and psychiatry) or surgical (general surgery, gynecology, neurosurgery, otorhinolaryngology and orthopedics) Specialties. The classification to pediatrics and ophthalmology is done by specialist physicians. This information is recorded in the reception sheet and stored in the information system of the institution.

The sample consisted of 611 digitized records of the patients over 18 years of age attended at the RRAC during the months of April to June 2014, as part of a master’s project approved under the CAEE: 05739412910015505. Inclusion criteria were all records of patients over the age of 18 attended in the proposed period. Incomplete or illegible records were excluded. Considering that this study was observational and the collection of patient data was done by means of electronic medical records, not causing any type of interference in the sector or on patient care, the study was exempted from the need to request informed consent forms, when the project was approved. Access to data took place through the Hospital Management System of the Information Technology Department - HSP, after authorization. The patient data analyzed were age, sex, comorbidities, duration of the complaint, medical specialty, signs and symptoms, outcome, color attributed in the risk classification and pain grade according to a numerical scale (NS) varying as follows: without pain (0); mild pain (1 - 4); moderate pain (5-7) and severe pain (8-10)^9^.

The software used for analysis was the Statistical Package for Social Sciences (SPSS), version 19. Descriptive analysis was used for sociodemographic characterization, color attributed in the risk classification, duration of the complaint and comorbidities. For the continuous variables, we calculated the mean, standard deviation, median, minimum and maximum and for categorical variables, frequencies and percentages. Analysis of Variance (ANOVA) was used to compare pain intensity with age. Pain intensity was correlated with sex, signs and symptoms, history of cancer, category of risk classification and medical specialty using the Chi-Square test and when necessary the Likelihood Ratio test. A significance level of 5% (p-value < 0.05) was adopted.

## Results

Among the 611 patient records analyzed in this study, the mean age was 42.1 (17.84) years; the majority were women 366 (59.9%); and the duration of complaint to receive care ranged from 1 to 365 days. Patients were classified in risk classification categories as follows: green (58.9%); yellow (22.7%); orange (7.9%); blue (5.9%) and red (4.6%). Most of them were attended by medical specialties of medical clinic (37.3%), orthopedics (16%) and surgery (13.4%); the majority was discharged (91.5%). The prevalent comorbidities were hypertension (18.3%) and diabetes mellitus (7.1%). The more prevalent symptoms were respiratory symptoms (14.4%) and pain (46.3%). Patients who were asked about pain reported no pain (37.6%), and mild (12.1%), moderate (25.9%) and intense (24.4%) pain.

Patients with no pain had a significantly higher age than those with moderate pain, with men presenting a higher percentage of absence of pain while women had more often intense pain (Table 1).

**Table 1:** Comparison of pain intensity according to age, sex, risk classification category and medical care specialty of the population studied. São Paulo, SP, Brazil, 2014

Variables	Intensity of pain				Total (100%) n	p-value
	Absence n (%)	Mild n (%)	Moderate n (%)	Severe n (%)		
Age (years)						
Mean (SD)*	44.7 (19.1)	41.3 (18.0)	37.8 (16.1)	42.9 (16.5)	42.1 (17.8)	0.0020^†^
Total	230	74	158	149	611	
Sex						
Female	125 (34.2)	37 (10.1)	100 (27.3)	104 (28.4)	366	0.0051^‡^
Male	105 (42.9)	37 (15.1)	58 (23.7)	45 (18.4)	245	
Total	230 (37.6)	74 (12.1)	158 (25.9)	149 (24.4)	611	
Classification						
Blue	16 (44.4)	14 (38.9)	3 (8.3)	3 (8.3)	36	<0.0001^‡^
Green	115 (32.1)	49 (13.7)	108 (30.2)	86 (24.0)	358	
Yellow	63 (45.7)	9 (6.5)	25 (18.1)	41 (29.7)	138	
Orange	18 (37.5)	1 (2.1)	14 (29.2)	15 (31.3)	48	
Red	15 (53.6)	1 (3.6)	8 (28.6)	4 (14.3)	28	
Total	227 (37.3)	74 (12.2)	158 (26.0)	149 (24.5)	608	
Specialty						
Cardiology	17 (65.4)	3 (11.5)	3 (11.5)	3 (11.5)	26	<0.0001^‡^
Surgery	22 (26.8)	12 (14.6)	17 (20.7)	31 (37.8)	82	
Medical clinic	101 (44.3)	23 (10.1)	60 (26.3)	44 (19.3)	228	
Gynecology	18 (36.0)	4 (8.0)	18 (36.0)	10 (20.0)	50	
Neurosurgery	5 (45.5)	-	4 (36.4)	2 (18.2)	11	
Neuroclinic	16 (55.2)	2 (6.9)	7 (24.1)	4 (13.8)	29	
Orthopedics	11 (11.2)	14 (14.3)	35 (35.7)	38 (38.8)	98	
ORL^§^	21 (31.8)	14 (21.2)	14 (21.2)	17 (25.8)	66	
Psychiatry	19 (90.5)	2 (9.5)	-	-	21	
Total	230 (37.6)	74 (12.1)	158 (25.9)	149 (24.4)	611	

*SD - Standard deviation; †Analysis of Variance; ‡Likelihood ratio; §ORL - Otorhinolaryngology

Patients classified in the red category had a higher percentage of absence of pain, whereas patients classified as green, yellow and orange had severe pain and those as blue had mild pain (Table 1).

Patients attended by psychiatry presented a higher percentage of absence of pain and those attended by orthopedics, of intense pain (Table 1).

In relation to signs and symptoms, patients with respiratory symptoms had a higher percentage of absence of pain, while those without these symptoms had a greater percentage of intense pain (Table 2).

**Table 2:** Comparison of pain intensity according to signs and symptoms presented by patients in the RRAC*. São Paulo, SP, Brazil, 2014

Variables	Intensity of pain				Total n (100%)	p-value
Symptom	Absence n (%)	Mild n (%)	Moderate n (%)	Severe n (%)		
Respiratory						
Yes	49 (55.7)	9 (10.2)	20 (22.7)	10 (11.4)	88	0.0008^†^
No	181 (34.6)	65 (12.4)	138 (26.4)	139 (26.6)	523	
Total	230 (37.6)	74 (12.1)	158 (25.9)	149 (24.4)	611	
IMPB^‡^						
Yes	12 (14.0)	12 (14.0)	31 (36.0)	31 (36.0)	86	<0.0001^†^
No	218 (41.5)	62 (11.8)	127 (24.2)	118 (22.5)	525	
Total	230 (37.6)	74 (12.1)	158 (25.9)	149 (24.4)	611	
Psychiatric						
Yes	22 (91.7)	2 (8.3)	-	-	24	<0.0001^†^
No	208 (35.4)	72 (12.3)	158 (26.9)	149 (25.4)	587	
Total	230 (37.6)	74 (12.1)	158 (25.9)	149 (24.4)	611	
Neurological						
Yes	30 (68.2)	2 (4.5)	6 (13.6)	6 (13.6)	44	0.0003^†^
No	200 (35.3)	72 (12.7)	152 (26.8)	143 (25.2)	567	
Total	230 (37.6)	74 (12.1)	158 (25.9)	149 (24.4)	611	
Nausea						
Yes	11 (26.8)	6 (14.6)	7 (17.1)	17 (41.5)	41	0.0427^†^
No	219 (38.4)	68 (11.9)	151 (26.5)	132 (23.2)	570	
Total	230 (37.6)	74 (12.1)	158 (25.9)	149 (24.4)	611	

*RRAC - Reception with Risk Assessment and Classification; †Chi-Square; ‡IMPB - Inability to move part of the body;

Patients who were not able to move their bodies and those with psychiatric and neurological symptoms had a higher percentage of absence of pain, while those with an inability to move part of the body had a higher percentage of moderate and intense pain (Table 2).

Patients who reported nausea had a higher percentage of intense pain, and those without nausea had a higher percentage of absence of pain and moderate pain (Table 2).

Patients with neoplasias (n = 25) had a higher percentage of absence of pain (n = 12, 48%) and intense pain (n = 10, 40%) (p = 0.0372).

## Discussion

The demand for urgent care has increased and most patients report pain at the time of RRAC. Based on this complaint, resources are used to classify and organize the priorities of these patients. Pain is one of the main reasons that can generate inabilities and cause human suffering, impacts the quality of life, and can generate psychosocial and economic repercussions^10^.

In this study, women predominated in the demand for Emergency Service in relation to men Some factors that may explain this is the resistance of men to seek health care due to societal taboos and gender-related sociocultural factors in which diseases are considered a sign of fragility and the search for medical services, a demonstration of weakness^11^.

In the risk classification categories, the majority of patients were classified as green (58.9%), followed by yellow (22.7%), orange (7.9%), blue (5.9% ) and red (4.6%), a similar result to another national study conducted in a public hospital in Diamantina, Minas Gerais, Brazil, in which low complexity patients were also the majority^12^. Thus, as already described in another study, one of the causes of overcrowding in ESs is the presence of less urgent cases that could be solved in primary health care^1^.

Hospital discharge (91.5%) was the most frequent outcome in this study, as well as of another study conducted in a public hospital in Minas Gerais that observed the relationship between risk classification, mortality and hospital stay. When the risk classification category attributed to patients was less severe, the chance of hospital discharge was greater^13^. This condition reinforces the need to strengthen managerial strategies in order to improve care according to the models of networked services.

As for comorbidities, hypertension (18.3%) and diabetes mellitus (7.1%) predominated, reflecting the high prevalence of these diseases in the general population^14^ ^-^ ^15^. It is known that these comorbidities are considered risk factors for several diseases, including cardiovascular diseases, and can subsequently cause important health problems, as well as an increased demand in Emergency Services^16^.

In the present study, the symptoms that most motivated the search for the service were pain (46.3%) and respiratory symptoms (14.4%). Similar results were found in two other national studies evaluating patients’ complaints according to the risk classification protocol^3^. The high percentage of complaints of pain may be related to the large number of patients who seek care in the specialty of medical clinic, mostly due to oncological and orthopedic diseases. Musculoskeletal diseases, that are a global health problem, commonly involve acute and chronic pain^17^.

Regarding the intensity of pain reported by the patients in this study, the majority reported no pain (37.6%), and the rest, moderate (25.9%), intense (24.4%) and mild (12.1%) pain. A similar study performed in a public hospital in Aracajú found prevalence of intense (53.7%) and moderate (36.6%) pain, concluding that pain intensity was related to the main reason for seeking emergency care^10^. Although pain is one of the main reasons for seeking emergency care and despite the existence of scales to assess its intensity, few professionals use these tools during care^7^. A study that evaluated the knowledge of nurses about pain showed that 73.3% never participated in a training on pain assessment and that their knowledge about pain management was medium^18^. This shows the importance of nurses to develop skills to make a complete evaluation of complaints, without underestimating the patients’ pain that can often be indicative of the severity of his health condition^10^.

Participants in this study who did not present pain were significantly older than those with moderate pain. A study carried out in an Emergency Service in Aracajú showed that patients with moderate pain complaints were younger than those who did not present pain, corroborating the results of the present study^10^. Studies show that age is a factor that may modify the experience of the patient regarding pain and the effect of aging may make them less sensitive to painful stimuli^19^.

Male patients had a higher percentage of absence of pain, while female patients had a higher percentage of intense pain. Pain is a personal and subjective experience, not only resulting from characteristics of tissue injury, but also integrating individual emotional and cultural factors^7^.

The patients in this study classified in the red category did not complain of pain. This is due in part to the fact that patients classified as red are at high risk of death and their pain assessment may be impaired due to prioritization of care, which is started even before the patient is registered in the hospital^(10, 12)^. Furthermore, it is common for severe patients to present altered consciousness, what prevents pain assessment^20^.

Patients classified as green, yellow and orange had a higher percentage of intense pain. The process of pain recognition informed at the moment of assessment is subjective and individual and may influence the risk category attributed to the patient^21^. In this study, patients classified in the green category reported severe pain. However, the existing classification protocols place higher intensity pain complaints as criteria of greater severity of the health status, because they generate physiological repercussions such as increased blood pressure, tachypnea, tachycardia, nausea, as observed in our study, among others. It is up to nurses to determine, by means of their perceptions, the proper category and waiting time to provide care for the patient^21^.

Pain is a common symptom presented in ESs. Although it is often underestimated, poorly assessed and treated, priority judgment often may not be appropriate in that situation. The nurses’ ability for not to interfere in the report of the pain intensity is still an obstacle to be faced. The application of a protocol for the adequate management of pain by the nurses can avoid delays in the treatment with analgesics and improve the quality of patient care^21^.

Patients attended by the medical specialty of psychiatry had a higher percentage of absence of pain. This result can be explained by the fact that pain evaluation involves the identification of the disease, etiologic factor, onset, duration, distribution, triggering and attenuating factors, quality and intensity of pain, as well as sensorial tests. In patients with an altered mental status, such condition may influence the evaluation of other signs and symptoms, requiring a differentiated assessment and individualized treatment, often difficult to perform in the ES due to the imminent risk situation^22^. Orthopedic patients had a higher percentage of intense pain; this result was already expected because musculoskeletal pain is the main cause of pain in the population^17^.

In this study, patients with respiratory symptoms had a higher percentage of absence of pain, while those who did not present respiratory symptoms presented a higher percentage of intense pain. Respiratory problems are frequent reasons for seeking ESs, are often not associated with significant pain symptoms; and determine situations of imminent risk of death. In these cases, the patients’ perception of pain may be impaired by respiratory discomfort and sometimes by the need for analgesia and sedation to obtain a patent airway and make it possible the use of mechanical ventilation^23^.

The absence of pain was more frequent in patients who did not present inability to move part of the body, while those with disability had a higher percentage of moderate and intense pain. Persistent pain and impaired mobility and function are conditions commonly associated with musculoskeletal problems. There is a close relationship between painful musculoskeletal conditions and inability or reduced ability to move or perform some kind of physical activity resulting in functional decline, loss of independence and poor quality of life. For these individuals, not only the usual analgesic treatment should be adopted, but also an individualized rehabilitation program^17^.

Patients with psychiatric and neurological symptoms presented higher percentage of absence pain while individuals without psychiatric and neurological symptoms presented moderate and intense pain. Physical, mental, psychological, behavioral and even social problems can play an important role in the perception of pain and the reactions before it, interfering in the central neuro-modulation of afferent stimuli. Different approaches, including psychological ones, have a great impact on the understanding and treatment of these individuals. Such patients often require further evaluation and therapy to obtain better results, as these disorders may exacerbate or adversely affect pain perception and therapeutic response^22^.

Patients with nausea presented, in most cases, severe pain. This finding may be related to the malaise that nausea causes, often leading to puke, causing the muscles in the abdominal wall and chest to contract, and consequently producing pain. These symptoms, too, may accompany a complex variety of gastrointestinal organic disorders and systemic diseases that may have pain as a consequence^24^. In addition, medications commonly used for pain control such as opioid analgesics often cause nausea as a side effect^25^.

Pain in cancer patients ranged from absence (48%) to severe (40%) intensity. This complaint may vary according to the stage of the disease, and studies show that 90% of patients in advanced stages of cancer feel more intense pain^24^. The control of cancer pain can be difficult and pain is often the final result of a process involving emotional, spiritual, cognitive and sensory aspects. Pain, in these cases, may be associated with disease progression and cause hopelessness and fear in the patients; these cases require a careful and differentiated approach to pain management^26^.

The limitations of this study were its realization in a single center, collection of data from medical records which were often incomplete and illegible and use of RRAC protocol with adaptations to the needs of the Institution limiting the comparison with other studies.

This study may contribute to practice insofar as its findings demonstrate the importance of professionals working in RRAC to be able to use pain intensity scales because this symptom is an individual and subjective experience, and its identification may influence the category of severity attributed and the experience of the patients regarding the quality of care received.

Since pain is one of the main reasons for seeking ESs, it is paramount that nurses have knowledge about it. In most cases, nurses are the professionals responsible for the first care measures and they define the flow of the patients in the service. All patients have the right to express their pain and receive treatment for this complaint, and the nurses and the multiprofessional team must implement effective strategies to relieve pain avoiding deleterious effects resulting from this symptom and providing patients with humanized care.

## Conclusion

In this study, the pain intensity reported by patients was most frequently of moderate intensity. The association of pain with the risk classification categories showed that patients classified in the red color showed, in most cases, absence of pain; those classified in the blue color had mild pain; and those classified in the green, yellow and orange colors had severe pain.
